# Evaluation of a remote monitoring app in head and neck cancer follow‐up care

**DOI:** 10.1002/cam4.6202

**Published:** 2023-06-09

**Authors:** Cecile van de Weerd, Tom Ebbers, Donna E. M. Smilde, Julia J. van Tol‐Geerdink, Robert P. Takes, Guido B. van den Broek, Rosella P. M. G. Hermens, Rudolf B. Kool

**Affiliations:** ^1^ Department of Otorhinolaryngology and Head and Neck Surgery Radboud University Medical Center Nijmegen the Netherlands; ^2^ Department of Radiation Oncology Radboud University Medical Center Nijmegen the Netherlands; ^3^ Department of IQ Healthcare Radboud University Medical Center Nijmegen the Netherlands

**Keywords:** aftercare, head and neck cancer, outpatient monitoring, qualitative research, telemedicine

## Abstract

**Background:**

A remote monitoring app was developed for head and neck cancer (HNC) follow‐up during the SARS‐CoV‐2 pandemic. This mixed‐methods study provides insight in the usability and patients' experiences with the app to develop recommendations for future use.

**Methods:**

Patients were invited to participate if they were treated for HNC, used the app at least once and were in clinical follow‐up. A subset was selected for semi‐structured interviews through purposive sampling considering gender and age. This study was conducted between September 2021–May 2022 at a Dutch university medical center.

**Results:**

135 of the 216 invited patients completed the questionnaire, resulting in a total mHealth usability score of 4.72 (± 1.13) out of 7. Thirteen semi‐structured interviews revealed 12 barriers and 11 facilitators. Most of them occurred at the level of the app itself. For example, patients received no feedback when all their answers were normal. The app made patients feel more responsible over their follow‐up, but could not fulfill the need for personal contact with the attending physician. Patients felt that the app could replace some of the outpatient follow‐up visits.

**Conclusions:**

Our app is user‐friendly, makes patients feel more in control and remote monitoring can reduce the frequency of outpatient follow‐up visits. The barriers that emerged must be resolved before the app can be used in regular HNC follow‐up. Future studies should investigate the appropriate ratio of remote monitoring to outpatient follow‐up visits and the cost‐effectiveness of remote monitoring in oncology care on a larger scale.

## 1 INTRODUCTION

Approximately 560.000 patients are diagnosed with head and neck cancer (HNC) each year worldwide.^1^ Follow‐up protocols prescribe clinical follow‐up for three years to lifelong after curative treatment, consisting of several outpatient follow‐up visits per year.^2^ Patients have stated that the current follow‐up regimen is intensive.^3^ Some report that the frequency of visits is excessive and that they prefer a less rigorous follow‐up schedule.^4^, ^5^ In addition, not all follow‐up visits are essential from a medical point of view—some are mainly intended to reassure the patient.^3^, ^6^, ^7^ Not only patients, but also healthcare professionals are willing to de‐intensify HNC follow‐up, provided that the doctor‐patient relationship is adequately maintained.^8^ Finally, the current follow‐up schedule pressures healthcare systems and contributes to high costs.^9^, ^10^

Using a remote monitoring application (RMA) within HNC follow‐up might actively engage patients in their care, optimize patient information delivery, reduce the number of required outpatient follow‐up visits and thereby reduce healthcare costs.^11^ RMAs are designed to collect patient‐entered data, such as patient‐reported outcome and experience measures, which can be received and interpreted by the hospital involved. We specifically developed an RMA for HNC patients during the SARS‐CoV‐2 pandemic and used it as an alternative to outpatient follow‐up visits since the pandemic led to a significant decrease in outpatient capacity at hospitals all over Europe.^12^ Our RMA remained available as an add‐on to regular follow‐up care when outpatient follow‐up visits were resumed.

Before further implementation into clinical practice, the RMA had to be evaluated and optimized. Therefore, this study aimed to investigate patients' experiences with remoting monitoring using our RMA during the SARS‐CoV‐2 pandemic. Our primary objectives were to evaluate the app's usability from a patient's perspective and to gain insight into the barriers and facilitators for using the RMA. In addition, we aimed to evaluate differences between patients over and under 65 years of age, as there is limited knowledge about the older oncology patient's experiences with and use of digital health services.^13^

## 2 METHODS

### 2.1 Study design and setting

This study used a mixed‐methods methodology, starting with a survey among HNC patients, followed by in‐depth interviews among a subset of these patients. This study was performed at the Radboud university medical center in Nijmegen, The Netherlands. This is one of the largest Dutch head and neck oncology centers, with approximately 500 newly diagnosed patients annually. The local ethics committee considered the study exempt from further review (dossier number 2020–6941). The consolidated criteria for reporting qualitative research (COREQ) checklist was used^14^ (Appendix A, Table A1).

### 2.2 Study population

Patients were eligible to participate in this study if they were treated for HNC and were currently in HNC follow‐up. According to the Dutch guidelines, this follow‐up consists of five years of prescheduled visits after treatment with decreasing frequency.^15^ Other criteria were that they were fluent in Dutch and 18 years or older. HNC patients who used the RMA at least once were invited to complete the mHealth App Usability Questionnaire (MAUQ), a validated questionnaire that objectively evaluated the usability of mHealth apps.^16^ Subsequently, qualitative interviews among a subset of participants were used to explore barriers and facilitators for using the RMA.

### 2.3 The remote monitoring application

**FIGURE 1:**
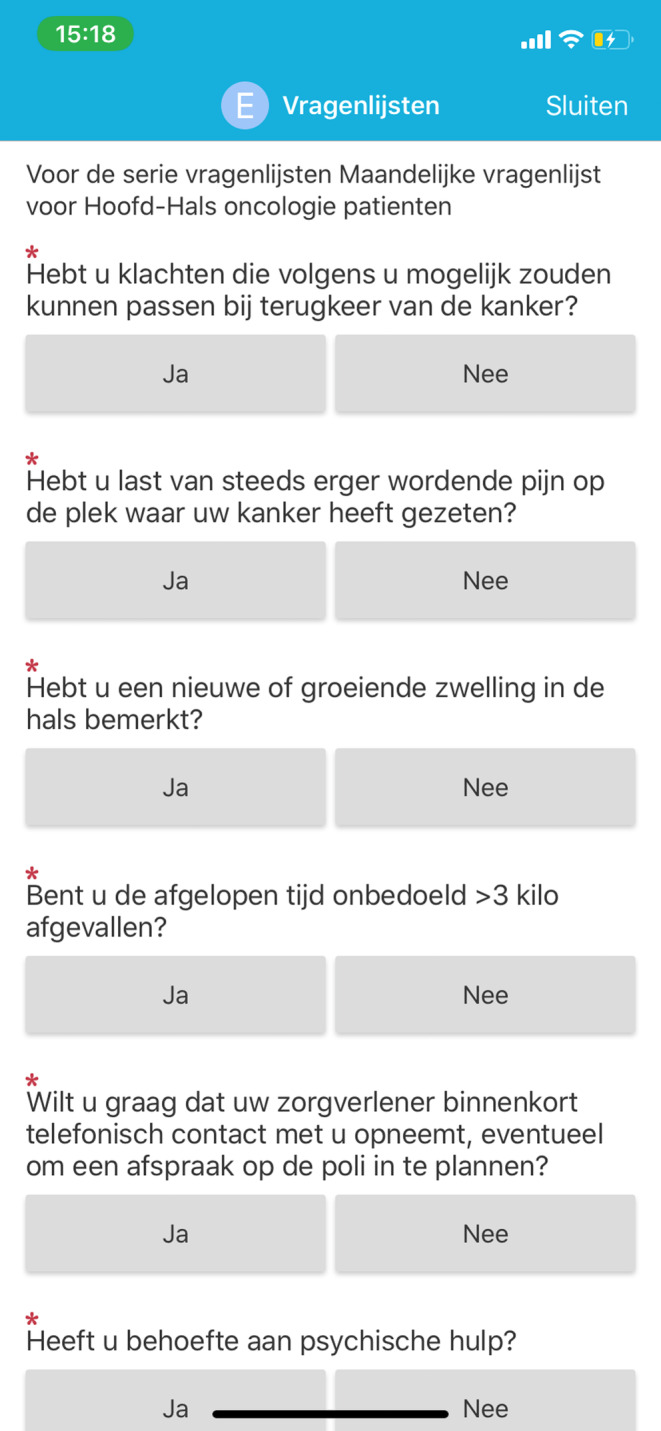
Screenshot of the remote monitoring application. Examples of the Dutch questions which the patient could answer with yes (“Ja”) or no (“Nee”).

**FIGURE 2:**
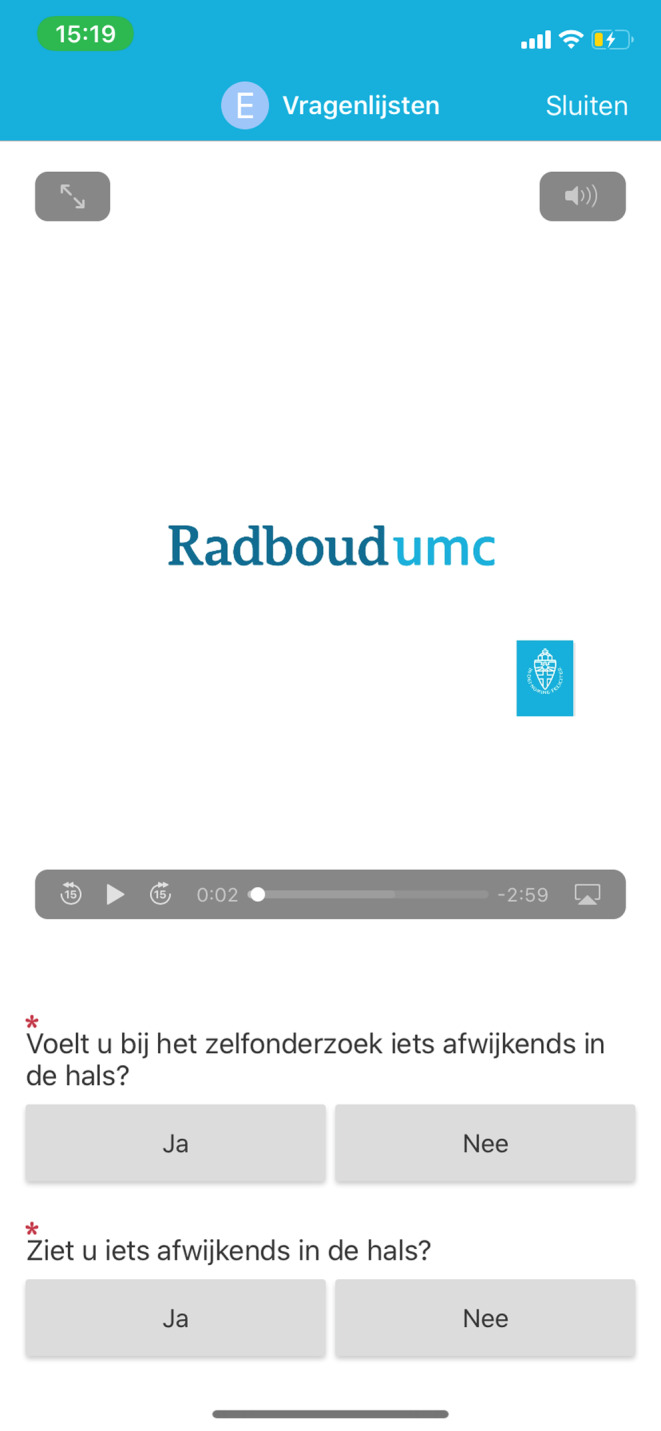
Screenshot of the remote monitoring application. A 3‐minute video was incorporated to explain self‐examination of the head and neck area. Patients were asked if they felt or saw anything abnormal.

Our RMA is designed to monitor HNC patients at home. The RMA was developed using EPIC MyChart (EPIC EHR, Verona, Wisconsin) and, therefore, integrated within the Electronic Health Record.^17^ Patients receive automatic monthly notifications for self‐monitoring, which is achieved by completing a short questionnaire and examining the head and neck area using video instructions by themselves or a relative. See Figures 1 and 2. Case managers, who are supportive healthcare professionals coordinating HNC care, are automatically notified by a message from the electronic health record in case of potential abnormal findings. These include that the patient experiences symptoms consistent with possible disease recurrence, requires psychosocial support, has a question, or uploaded a photo or video that needs to be checked. The case manager reviews the patients' results, and decides whether the treating physician needs to be consulted and if the patient needs to visit the outpatient clinic.

### 2.4 Data collection

#### 2.4.1 mHealth app usability questionnaire

The MAUQ consists of 21 questions and was developed to evaluate the usability of a mobile health app by statements on ease of use and satisfaction, system information arrangement and usefulness, which can be answered using a 7‐point Likert scale (strongly disagree—strongly agree).^16^ To translate the English version of the MAUQ to Dutch, translation guidelines for validated questionnaires were followed. Multiple forward‐ and backward translations were conducted by two independent professional translators from the Radboud University, department Radboud in'to Languages. Discrepancies were discussed and resolved by previous mentioned translators, TE, RK, and GB.^18^ The final version of the Dutch MAUQ and the original English version are included in Appendix A, Methods A1 and A2. All eligible patients received an invitation to complete the MAUQ through CastorEDC, an electronic data capture platform.^19^ After completing the MAUQ, patients were asked if researchers could contact them for an in‐depth interview. The quantitative data was collected between September and November 2021. All answers were processed anonymously.

#### 2.4.2 Interviews

Patients were selected through purposive sampling based on gender and age from the population who completed the MAUQ and agreed to participate in the interview.^20^ A semi‐structured interview guide was developed by two researchers (CW, TE) based on literature published about usability of mHealth applications and the questionnaire results. Two experts in qualitative research (RK, RH) reviewed the interview guide. The interview guide covered four topics: use of the RMA, content of the RMA, influence of remote monitoring on perceived care, and future perspectives on remote monitoring in HNC care. Questions were open‐ended and were optionally followed by questions to expand on each topic. See Appendix A, Methods A3, for the interview guide.

After two interviews, three researchers (CW, DS, TE) reviewed the interview guide and made minor adjustments accordingly. Interviews were conducted until data saturation, the point at which no new information was mentioned in the interviews, was reached.^21^

Two researchers (CW, DS) were trained in interviewing and conducted the interviews in January and February 2022. No one was present besides the interviewer(s) and the participant. Participants were informed about the role of the researcher and the study goals. There was no relationship between the participants and the researcher, nor did the researcher benefit from certain outcomes. Written and verbal informed consent was achieved before each interview. Participation was voluntary, and patients could withdraw from the study at any moment without consequences.

### 2.5 Data analysis

#### 2.5.1 mHealth app usability questionnaire

The mean and standard deviation (SD) for each subscale and the total usability score were calculated according to the MAUQ instruction guide.^16^ A Mann–Whitney‐U test was used to compare the total scores of patients under 65 to those of 65 years and above. Two‐sided *p*‐values of <0.05 were considered statistically significant. IBM SPSS Statistics for Windows, version 25 (IBM Corp., Armonk, N.Y., USA) was used for all analyses.

#### 2.5.2 Interviews

Thematic analysis as described by Braun & Clarke in 2006 was used as follows to analyze the data that emerged from the interviews. The interviews were recorded to be anonymously transcribed by an independent third‐party company. Participants did not receive the transcripts for corrections because they were transcribed verbatim. Two researchers (CW, DS) read and labeled the interviews by open‐ended coding using Atlas.ti (Scientific Software Development GmbH, Berlin, Version 9). Coding differences were discussed by four members of our team (CW, DS, TE, RK) until consensus was reached. There were three moments of reflection during the coding process. The codes were compiled into subthemes. Two researchers (CW, DS) merged these subthemes into the six levels of the barriers and facilitators framework published by Grol and Wensing.^22^ Again, differences were discussed until a consensus was reached. Afterwards, results were discussed with a fifth researcher (RH) and adjusted accordingly.

## 3 RESULTS

### 3.1 Participants

In total, 216 patients who at least used the RMA once were invited to complete the MAUQ. After three reminders, 135 patients completed the questionnaire, resulting in a 63% response rate. The mean respondent age was 66.2 (± 9.7) years. Thirteen semi‐structured interviews were conducted for the qualitative part of this study. The interviewees' ages ranged from 54 to 71 years, with a mean of 63.8 (± 5.0). The majority (*n* = 8) were male. Other participant characteristics are shown in Table 1.

**TABLE 1:** Participant characteristics.

Questionnaires (*n* = 135)						
Age, years Mean (SD)	66.2 (± 9.7)					
Gender No. (%)	Male 85 (63)	Female 50 (37)				
Interviewees (*n* = 13)	Age, years	Gender	Educational level	Tumor localization	Treatment	Follow‐up year
Patient 1	60	Male	Intermediate	Oropharynx	Radiotherapy	5
Patient 2	65	Female	Basic	Larynx	Radiotherapy	5
Patient 3	68	Male	Advanced	Oropharynx	Chemotherapy + radiotherapy	3
Patient 4	66	Male	Intermediate	Oral cavity	Surgery + chemotherapy + radiotherapy	2
Patient 5	61	Female	Basic	Oral cavity	Surgery + chemotherapy + radiotherapy	5
Patient 6	62	Female	Advanced	Nasal cavity	Surgery + chemotherapy + radiotherapy	2
Patient 7	64	Male	Intermediate	Oropharynx	Chemotherapy + radiotherapy	3
Patient 8	71	Male	Basic	Hypopharynx	Chemotherapy + radiotherapy	2
Patient 9	65	Male	Advanced	Oropharynx	Chemotherapy + radiotherapy	3
Patient 10	54	Female	Basic	Oral cavity	Surgery	2
Patient 11	65	Male	Advanced	Oral cavity	Surgery + radiotherapy	3
Patient 12	71	Female	Basic	Oral cavity	Surgery	2
Patient 13	57	Male	Intermediate	Oropharynx	Chemotherapy + radiotherapy	2

### 3.2 mHealth app usability questionnaire

The total mHealth usability score as measured by the MAUQ was 4.72 (± 1.13) on a scale from 1 to 7. The subscale scores for ease of use and satisfaction, system information arrangement, and usefulness were 5.0 (± 1.23), 4.74 (± 1.15), and 4.42 (± 1.24), respectively. The statements “the app was easy to use” and “it was easy to learn to use the app for me” scored the highest: 5.39 and 5.47, respectively. Patients also felt confident that the RMA sent the information they entered to their healthcare provider (score: 5.27). The statements “I had many more opportunities to interact with my healthcare provider” and “the app helped me manage my health effectively” scored the lowest: 4.06 and 4.15, respectively. The results of all statements are shown in Table 2. Patients younger than 65 scored 4.63 (± 1.16) and patients of 65 years or older scored 4.72 (± 1.20). There was no significant difference between the two groups (*p* = 0.627).

**TABLE 2:** Results of the mHealth Usability Questionnaire (MAUQ).

MAUQ subscales and corresponding statements^a^	Mean	(SD)
**1. Ease of use and satisfaction score (MAUQ_E)**	**5.00**	**± 1.23**
1. The app was easy to use.	5.39	± 1.30
2. It was easy for me to learn to use the app.	5.47	± 1.29
3. I like the interface of the app.	4.81	± 1.46
4. The information in the app was well organized, so I could easily find the information I needed.	5.19	± 1.19
5. I feel comfortable using this app in social settings.	4.56	± 1.59
6. The amount of time involved in using this app has been fitting for me.	5.15	± 1.33
7. I would use this app again.	4.53	± 1.77
8. Overall, I am satisfied with this app.	4.91	± 1.59
**2. System information arrangement score (MAUQ_S)**	**4.74**	**± 1.15**
9. Whenever I made a mistake using the app, I could recover easily and quickly.	5.00	± 1.25
10. This mHealth app provides an acceptable way to receive healthcare services.	4.43	± 1.68
11. The app adequately acknowledged and provided information to let me know the progress of my action.	4.79	± 1.44
12. The navigation was consistent when moving between screens.	4.86	± 1.27
13. The interface of the app allowed me to use all the functions (such as entering information, responding to reminders, viewing information) offered by the app.	4.66	± 1.25
14. This app has all the functions and capabilities I expected it to have.	4.71	± 1.34
**3. Usefulness score (MAUQ_U)**	**4.42**	**± 1.24**
15. The app would be useful for my health and well‐being.	4.55	± 1.61
16. The app improved my access to healthcare services.	4.21	± 1.51
17. The app helped me manage my health effectively.	4.15	± 1.45
18. The app made it convenient for me to communicate with my healthcare provider.	4.26	± 1.49
19. Using the app, I had many more opportunities to interact with my healthcare provider.	4.06	± 1.45
20. I felt confident that any information I sent to my healthcare provider using the app would be received.	5.27	± 1.29
21. I felt comfortable communicating with my healthcare provider using the app.	4.45	± 1.44
**Total score**	**4.72**	**± 1.13**

^a^ 1—strongly disagree; 2—disagree; 3—somewhat disagree; 4—neither agree nor disagree; 5—somewhat agree; 6—agree; 7—strongly agree.

### 3.3 Interviews

Due to the SARS‐CoV‐2 regulations, interviews were performed by telephone, with a mean duration of 31 minutes (range 16–51). Data saturation was reached after 11 of the 13 interviews. A total of 12 barriers and 11 facilitators emerged and were classified into five levels of Grol and Wensing's framework. The innovation level covered nine of the 23 barriers and facilitators.^22^ No barriers or facilitators fit the economic and political context, and this level was, therefore, omitted. The results are shown in Table 3.

**TABLE 3:** Barriers and facilitators that promote or inhibit use of the RMA for patients, according to Grol & Wensing's framework.

Level	Barrier	Facilitator
Innovation	The patient receives no automatic feedback when results are negative The RMA cannot confirm the truth of patients' answers The instruction video for self‐examination cannot be saved or rewound The instruction video for self‐examination is not equally applicable for every type of HNC The RMA lacks a free‐text field to clarify answers to the questions in more detail	The RMA can extend the time between hospital visits The RMA is easy to use The RMA consists of straightforward questions aimed at detecting possible cancer recurrence Filling out the RMA is less time‐consuming than a hospital visit
Individual professional	Patients experience a lack of explanation from their physician regarding the interpretation of self‐examination of the head and neck area	The physician shows a clear explanation in the video on how to conduct self‐examination of the head and neck
Patients	Patients are unnecessarily occupied with illness Patients experience a lack of confidence to conduct self‐examination Patients experience a lack of knowledge to interpret their findings during self‐examination	Patients do not need to learn new things to use the RMA Patients are more alert to symptoms of possible cancer recurrence when using the RMA The RMA increases patients' self‐responsibility within follow‐up
Social context	Patients need help from others in performing self‐examination The RMA cannot fulfill the need for personal contact with the treating physician	Patients feel more connected to the hospital
Organizational context	The RMA was not properly introduced to patients Endoscopic examination cannot be done using the RMA	Patient can have easy and quick contact with the hospital when needed Using the RMA can take place at the patient's preferred time

#### 3.3.1 Innovation

The interviewees indicated that the RMA was easy to use. The questions were straightforward and focused on detecting possible cancer recurrence. However, patients would like to provide more nuanced answers and suggested adding a free‐text field to elaborate on the answer or having more answer options than “yes” and “no.”

‘The questions are fully focused on my situation, on throat cancer. I think they are adequate.’—Patient #9.

Interviewees encountered some problems while conducting self‐examination of the head and neck. The instruction video could not be saved or rewound. In addition, some interviewees pointed out that the self‐examination explained in the video did not specifically address investigation of their tumor localization.

‘You cannot save the video or watch it again.' So, you cannot stop the video and be like, “what should I do now?”—Patient #4.

‘The video only demonstrates how to examine the outside area. My type of cancer was in my mouth, and sometimes I thought, “should I not look in my mouth?” […] Not everyone has the same thing, it is very personal.’—Patient #7.

An advantage of using the RMA was that it is less time‐consuming than a hospital visit. Interviewees also stated that follow‐up by the RMA could reduce the frequency of hospital visits by extending the time between visits. When patients answer “yes” to questions within the RMA, a notification reaches the case manager, who contacts the patient to verify the results. When patients answer “no” to each question, all results are normal, and they receive no feedback from the RMA. Patients who always had a normal result indicated that they were unaware of what happened with their data.

‘So, if I enter “no” in every question, I'll get “You have completed all your assignments again”, but I won't get, after two days, “We've looked into it, glad it's going so well”.’—Patient #3.

#### 3.3.2 Individual professional

Healthcare professionals clearly demonstrated how to self‐examine the head and neck area in the video. However, patients also reported that an explanation of interpreting the findings was lacking.

‘If they had explained something in the video like “look for a bump or look for hard parts or whatever”, it would have been clearer. The video does explain very clearly where to examine, but not what to examine.’—Patient #7.

#### 3.3.3 Patient

Patients reported not having to learn new things to use the RMA. Besides, the RMA allowed patients to be more involved with their illness and symptoms. Patients felt more self‐responsibility when using the RMA compared to outpatient hospital visits. Some considered this positive, as it gave them more control over their follow‐up. Others reported a negative effect, as they sometimes felt unnecessarily preoccupied with their symptoms.

‘The RMA signals: listen up, you need to check yourself. Because the RMA reminds me of that, I also sometimes perform the self‐examination when I sit quietly for a while, without having received a notification from the RMA.’—Patient #7.

‘Imagine; it's beautiful weather and you go for a walk. You get home, and you get that notification again. You have to do another one of those examinations. Then I feel all kinds of things again. It makes me insecure.’—Patient #4.

One barrier mentioned was the difference in how physicians and patients examine the head and neck. Some patients expressed less confidence and knowledge in their way of examining than that of a physician.

‘The doctors know what to feel, but I don't. I don't feel that, I don't know what to feel, what to look for. They do explain it in the video, but I can't do anything with that.’—Patient #2.

#### 3.3.4 Social context

Some patients reported needing help from a relative to complete the self‐examination as a barrier to using the RMA. A facilitator was that the RMA made patients feel more connected to the hospital.

“I do think it's a good thing to keep using the RMA, because it also reminds people again of: “the hospital is still thinking about me, they read the answers I give and want to know how I am doing”.—Patient #12.

Patients reported missing personal contact with healthcare professionals when using the RMA. The RMA could not offer the personal attention that they experienced during a physical visit.

‘I do miss being able to go back to the hospital. They call and then they ask, “How are you?”, and that's it. I can examine my neck, but I don't think that's the solution.’—Patient #8.

#### 3.3.5 Organizational context

The RMA made it easier for patients to contact the hospital when needed. Patients also stated that the RMA allowed them to monitor their complaints at their preferred time.

‘I can complete the RMA at the time I prefer. The notification can come in spontaneously sometime during the day. Once I have seen it, I will fill it out in the evening when I have the time.’—Patient #7.

The RMA was introduced during the SARS‐CoV‐2 pandemic. Patients received an email with information about the RMA. According to some patients, this introduction was incomplete and unclear. Some patients reported the lack of flexible endoscopic examination as a shortcoming.

## 4 DISCUSSION

This mixed‐method study provides insight into the usability of an RMA developed by a Dutch university medical center during the SARS‐CoV‐2 pandemic and patients' experiences with this RMA in HNC follow‐up care. The overall usability of the RMA assessed with the mHealth Usability Questionnaire was good, particularly in terms of ease of use. The fact that there were no significant differences in MAUQ scores for patients over and under 65 years of age supports the assumption that our RMA is suitable for patients of all ages. Semi‐structured interviews revealed 12 barriers and 11 facilitators for use in daily practice from patients' perspectives. Self‐responsibility and the ability to perform checkups at their preferred time were mentioned as facilitators of using the RMA. Barriers included the interpretation of self‐examination of the head and neck area and the lack of personal contact with their treating physician.

Patients felt that the RMA provided quick communication with their healthcare providers in case they filled out that they were experiencing physical symptoms. Van den Brink et al. also concluded that an electronic health system allowed for early detection of problems in HNC care that required direct intervention.^23^ They hypothesized these problems could have led to adverse events had they been discovered during later outpatient visits. This could also be the case in our study, although we did not review individual reported symptoms. The MAUQ showed that patients were confident that information sent through the RMA would be received by their healthcare providers. The interviews revealed that patients received no feedback from the RMA in case all their answers were normal. During the development process of the RMA, we chose to only review abnormal patient answers to keep the automated monitoring process as efficient as possible. An explanation on when healthcare providers will review answers should be added to the RMA to better inform the patient.

The MAUQ did not cover all topics that emerged from the interviews. First, patients reported that the RMA made them more alert to symptoms of possible cancer recurrence in daily life and gave them a higher sense of self‐responsibility. Bouaoud et al. also concluded that mHealth applications enable the early detection of health problems and improve HNC patients' self‐management.^24^ While our patients felt the instructions on examining the head and neck area were clear, directions on interpreting their findings were perceived as insufficient. A solution might be to practice the self‐examination with a healthcare provider before starting remote monitoring to increase patients' confidence in distinguishing normal from abnormal findings. However, Addeo et al. described a number of reasons for nonoptimal patient communication, including a lack of time or staff.^25^ Time for patient education should, therefore, be scheduled. Also, the reliability of patient‐examination in comparison to physician‐examination could be studied in the future. Second, some patients clarified that the RMA did not fully apply to every HNC localization. For example, the instruction video does not cover examining the oral cavity. Duman‐Lubberding et al. also reported that patients felt that Oncokompas, an e‐health application for self‐management after cancer treatment, was not tailored to their individual needs.^26^ Our RMA could be personalized more by adding specific instruction videos for various HNC localizations. The RMA's complexity should be considered, as this could negatively affect the overall usability. Finally, patients felt that remote monitoring could not replace personal contact with their treating physician. Chen et al. reported clinicians' willingness to de‐intensify HNC surveillance and also expressed the importance of maintaining the patient‐physician relationship.^8^ Therefore, we suggest that our RMA could be used to reduce the frequency of routine visits in individualized follow‐up care, but not fully replace outpatient visits. Furthermore, shared decision‐making on whether or not to use this tool in follow‐up is essential because some patients could benefit significantly from the RMA, while others could be negatively affected due to increased preoccupation with their disease. As discussed by D'Allessandro et al., there is no “universal choice of treatment,” or in our case, follow‐up.^27^ Future studies should also focus on integrating remote monitoring into HNC follow‐up care while adequately maintaining the patient‐physician relationship, and meeting the need for supportive care for cancer patients. Another aspect that needs further investigation is the cost‐effectiveness of integrating remote monitoring in cancer follow‐up care.

The main strength of this study is the mixed‐methods design. Other studies have also investigated the feasibility and usability of remote monitoring in head and neck oncology using quantitative methods.^28^, ^29^ The results showed that remote monitoring of symptoms is feasible and that the applications were useful, which is consistent with our findings. However, through our qualitative analysis, additional barriers and facilitators were found, which can be used to optimize our RMA further.

This study also has some limitations. We cannot completely exclude selection bias. Patients with a higher educational level or more affinity with technology could be more likely to use the RMA and more inclined to participate in this study. One patient who had used the RMA once decided not to continue it because he was not satisfied with it. Unfortunately, this patient was not willing to participate in this study. There may be more patients who feel the same way. It would be interesting to study patients who chose not to start or continue using the RMA to understand the barriers for use better. Unfortunately, patients who started using the RMA during the pandemic did not give consent for participation in a research study nor for researchers to extract information from their medical records, making it difficult to compare patients who used the RMA with a general HNC population. Also, we did not have access to detailed demographic and clinical data of patients that solely agreed to complete the MAUQ because of privacy reasons. Therefore, we could not investigate whether certain characteristics such as HNC‐site or ‐stage were related to differences in MAUQ scores. Finally, the response to the MAUQ was 63% after sending thee reminders. One could argue that a considerable amount of patients did not respond. However, our response rate was higher than the average email‐survey response rate of 51% among surgical patients recently described in a systematic review by Meyer et al.^30^

It should be noted that our RMA focuses on changes in physical function that may indicate recurrence. Although disease surveillance is one goal of cancer follow‐up care, monitoring functional and psychosocial status is also important. Dutch HNC patients routinely receive symptom‐related questionnaires focusing on these domains through the Dutch Head and Neck Audit.^31^ Our RMA and this study focus on detecting recurrence‐related symptoms.

## 5 CONCLUSION

This study shows that our RMA is user‐friendly, making patients feel more connected to the hospital and more alert to new symptoms. The RMA can be improved by following patient suggestions, such as explaining how to interpret self‐examination of the head and neck and giving patients feedback when the monitoring results are normal. Patients indicate that remote monitoring could reduce the frequency of outpatient visits, providing that the physician‐patient relationship is adequately maintained. As such, the RMA could help to relieve the pressure on HNC follow‐up care in the future. Since detecting recurrences or second primary tumors is an important goal of HNC follow‐up, the next step would be to study the effectiveness of disease detection through remote monitoring.

## AUTHOR CONTRIBUTIONS

**Cecile van de Weerd:** Conceptualization (equal); data curation (equal); formal analysis (equal); investigation (equal); project administration (equal); visualization (equal); writing – original draft (equal). **Tom Ebbers:** Conceptualization (equal); data curation (equal); formal analysis (equal); investigation (equal); project administration (equal); software (equal); writing – original draft (equal). **Donna E.M. Smilde:** Data curation (equal); formal analysis (equal); investigation (equal); writing – original draft (equal). **Julia van Tol‐Geerdink:** Conceptualization (equal); supervision (equal); writing – review and editing (equal). **Robert Takes:** Conceptualization (equal); project administration (equal); supervision (equal); writing – review and editing (equal). **Guido B. van den Broek:** Conceptualization (equal); project administration (equal); software (equal); supervision (equal); writing – review and editing (equal). **Rosella P.M.G. Hermens:** Conceptualization (equal); project administration (equal); supervision (equal); writing – review and editing (equal). **Rudolf B. Kool:** Conceptualization (equal); project administration (equal); supervision (equal); writing – review and editing (equal).

## FUNDING INFORMATION

This research did not receive any specific grant from funding agencies in the public, commercial, or not‐for‐profit sectors.

## CONFLICT OF INTEREST STATEMENT

The authors declare that they have no known competing financial interests or personal relationships that could have appeared to influence the work reported in this paper.

## PRECIS

Remote monitoring with an app makes head and neck cancer patients feel more in control over their follow‐up and could reduce the frequency of outpatient follow‐up visits.

## Data Availability

The data that support the findings of this study are available from the corresponding author, CW, upon reasonable request.

## APPENDIX A

### A.1 Methods A1. Original English mHealth App Usability Questionnaire (MAUQ)

**Ease of use and satisfaction score (MAUQ_E)**

1. The app was easy to use.
2. It was easy for me to learn to use the app.
3. I like the interface of the app.
4. The information in the app was well organized, so I could easily find the information I needed.
5. I feel comfortable using this app in social settings.
6. The amount of time involved in using this app has been fitting for me.
7. I would use this app again.
8. Overall, I am satisfied with this app.

**System information arrangement score (MAUQ_S)**

9 Whenever I made a mistake using the app, I could recover easily and quickly.
10 This mHealth app provides an acceptable way to receive healthcare services.
11 The app adequately acknowledged and provided information to let me know the progress of my action.
12 The navigation was consistent when moving between screens.
13 The interface of the app allowed me to use all the functions (such as entering information, responding to reminders, viewing information) offered by the app.
14 This app has all the functions and capabilities I expected it to have.

**Usefulness score (MAUQ_U)**

15 The app would be useful for my health and well‐being.
16 The app improved my access to healthcare services.
17 The app helped me manage my health effectively.
18 The app made it convenient for me to communicate with my healthcare provider.
19 Using the app, I had many more opportunities to interact with my healthcare provider.
20 I felt confident that any information I sent to my healthcare provider using the app would be received.
21 I felt comfortable communicating with my healthcare provider using the app.

### A.2 Methods A2. Translated Dutch mHealth App Usability Questionnaire (MAUQ)

**Gebruiksgemak en tevredenheid (MAUQ_E)**

1. De app was eenvoudig te gebruiken.
2. Het gebruik van de app was eenvoudig te leren.
3. De gebruikersinterface van de app bevalt me.
4. De informatie in de app was overzichtelijk, dus ik kon de informatie die ik nodig had gemakkelijk vinden.
5. Ik voel me op mijn gemak als ik de app in het bijzijn van anderen gebruik.
6. De hoeveelheid tijd die benodigd is voor het gebruik van deze app was passend voor mij.
7. Ik zou deze app nogmaals gebruiken.
8. In het algemeen ben ik tevreden over deze app.

**Organisatie van systeeminformatie (MAUQ_S)**

9 Als ik tijdens het gebruik van de app een fout maakte, kon ik die eenvoudig en snel herstellen.
10 Deze app was een geschikte manier om zorg te ontvangen.
11 De app bevestigde mijn invoer adequaat en gaf op heldere wijze informatie over de voortgang van mijn handeling.
12 De navigatie was consistent bij het wisselen tussen schermen.
13 Met de gebruikersinterface van de app kon ik alle functies gebruiken (zoals informatie invoeren, op herinneringen reageren, informatie bekijken) die de app biedt.
14 Deze app beschikt over alle functies en mogelijkheden die ik ervan verwacht.

**Nut (MAUQ_U)**

15 De app zou nuttig zijn voor mijn gezondheid en welzijn.
16 De app verbeterde mijn toegang tot de zorg
17 Met de app kon ik effectief aan mijn gezondheid werken.
18 De app maakte het gemakkelijk voor mij om met mijn zorgverlener te communiceren.
19 Dankzij het gebruik van de app had ik veel meer mogelijkheden tot interactie met mijn zorgverlener.
20 Ik had er vertrouwen in dat alle informatie die ik via de app naar mijn zorgverlener verstuurde ontvangen zou worden.
21 Ik voelde me op mijn gemak om via de app met mijn zorgverlener te communiceren.

### A.3 Methods A3. Interview guide (translated from Dutch)

**Introduction**

- Can you remember when you first used our remote monitoring app?
- Do you still use the remote monitoring app?

**Theme 1: Use of the app**

Overall, what did you think of using the remote monitoring app?

- What was easy when using the app? Why?
- What was more difficult when using the app? Why?
- Did you have to learn new things before you could use the app? What things?
- Did you need help from anyone else to use the app? At what?
- Did you run into any problems while using the app? Which ones? Did you manage to solve them? How?

**Theme 2: Content of the app**

What do you think of the questions asked in the app?

- What do you think of the clarity of the questions? What was good? What could be better?
- Were there any questions that were difficult to complete? Which ones? Why?
- Do you have any suggestions for improvement of the questions? If so, which ones?

What do you think of the explanation of the self‐examination of the neck in the app?

- Was the explanation clear? What was good? What could be better?
- Did the video help you? How? What was good? What could be better?
- Did you manage to perform self‐examination? Did you do this by yourself or with someone else? Why?
- Do you have any suggestions for improvement of the self‐examination section? If so, which ones?

The app has a link to Oncokompas at the end. Did you click on it?

- If yes: are you using Oncokompas due to this link? Why yes/no?
- Do you think the link to Oncokompas is of added value to the remote monitoring app? Why?

**Theme 3: Influence of remote monitoring on perceived care**

What do you think of remote monitoring with the app compared to standard follow‐up visits at the hospital?

- What do you think are the advantages of remote monitoring with the app compared to hospital checkups? Why?
- What do you think are the disadvantages of remote monitoring with the app compared to hospital checkups? Why?
- What do you miss in remote monitoring with the app compared to hospital checkups? Why?
- Do you trust remote monitoring with the app? Why?
- Has anyone from the hospital ever contacted you because of your answers in the app? What happened? What did you think of that?
- What do you think of the help the hospital offered in using the Home Monitor app? What was good? What could be better?
- How do you feel when you use the app? (Anxious, reassured, etc.) Does this differ from follow‐up visits at the hospital?
- Does the app affect how often you are reminded of your illness? Why?

**Theme 4: Remote monitoring in the future**

What do you think of the app being used more often in the future?

- How would we best introduce the app to patients? And when?
- What do you think of being monitored with the app instead of standard hospital follow‐up visits? Why?
- What do you think of a combination of follow‐up visits in the hospital and follow‐up with the app?
- Would you recommend the app to other patients? Why?
- Are there any areas of improvement for the app that we have not yet discussed? If so, which ones?
- What would you have wanted to know before you started using the app?
- What would you tell a patient who was about to start using the app?
- What would the ideal aftercare look like for you? And what role could the app play in that?

**Wrapping up**

- Are there any topics that we have not discussed yet that are of importance to you?
- Do you have any remaining questions?

**TABLE A1:** COREQ‐checklist.

Topic	Item No.	Description	Reported on page No. or not applicable (N/A)
**Domain 1: Research team and reflexivity**			
Personal Characteristics			
Interviewer/facilitator	1	The interviews were conducted by one or two researchers (DS, CW).	10
Credentials	2	Three researchers (CW, TE, DS) were trained in conducting qualitative health research prior to this study. Two researchers had conducted qualitative health research before (RT, GB). Two researchers (RH, TK) have extensive experience in conducting qualitative health research.	9
Occupation	3	Two researchers (CW, TE) are PhD‐students at the Radboudumc. One researcher (DS) is a master student in medicine. One researchers (TK) is a senior researcher at the department IQ healthcare at the Radboudumc. One researcher is a senior researcher at the department of Radiation Oncology at the Radboudumc (JT). Two researchers are ENT−/head and neck surgeon at the Radboudumc (RT, GB). One researcher is professor in head and neck oncology (RT), one researcher is professor in personalized medicine (RH).	N/A
Gender	4	The research team consists of 4 men and 4 women.	N/A
Experience and training	5	All researchers were trained or experienced in conducting qualitative research.	10
Relationship with participants			
Relationship established	6	The interviewers had no relationship with the study participants.	10
Participant knowledge of the interviewer	7	Participants were informed of the employment of the researchers, the role of the researcher in this study and the goal of the study (optimizing head and neck cancer follow‐up care).	10
Interviewer characteristics	8	The interviewers were trained in conducting semi‐structured interviews. They were aware of the literature on the research topic.	10
**Domain 2: Study design**			
Theoretical framework			
Methodological orientation and Theory		Thematic analyses were used. Interview results were analyzed using open‐ended coding. The barriers and facilitators framework of Grol and Wensing was applied to categorize the subthemes into 5 levels.	11, 14
Participant selection			
Sampling		Purposive sampling was used to select patients from the study population for the interviews considering gender and age.	9
Method of approach		Patients who at least used the RMA once were invited to fill out the MAUQ questionnaire. After completing the MAUQ, they were asked if they could be contacted for an in‐depth interview.	8
Sample size		13 patients were interviewed.	Result section
Nonparticipation		Several potential participants did not respond to the invitation to participate. They did not report a reason. One participant particularly stated that he did not want to be interviewed because the RMA was not useful for him.	17
Setting			
Setting of data collection		All interviews were conducted by telephone.	10
Presence of nonparticipants		No one was present besides the interviewer(s) and the participant.	10
Description of sample		A description of the sample is shown in the results section.	Table 2
Data collection			
Interview guide		A semi‐structured interview guide was developed by two researchers (CW, TE) based on relevant literature, whereafter two researchers who are expert in qualitative research (RH, TK) reviewed and adjusted it.	9, 10
Repeat interviews		None	N/A
Audio/visual recording		The interviews were audio‐recorded to be transcribed. The audio recording was turned on after the participant's approval for the recording.	10, 11
Field notes		None	N/A
Duration		The average interview duration was 31 minutes.	11
Data saturation		Interviews were conducted until data saturation (the point at which no new information was mentioned in the interviews) was reached.	10
Transcripts returned		Transcripts were not returned to participants for comments and/or corrections.	10
**Domain 3: analysis and findings**			
Number of data coders		Coding was performed individually by two researchers (CW, DS). There were several moments) of discussion and reflection of the codes between the researchers (CW, DS, TE, TK, RH).	11
Description of the coding tree		The subthemes were merged into 5 levels of the framework of Grol and Wensing.	11
Derivation of themes		Subthemes were derived from the data. These were categorized into themes from the Grol and Wensing framework.	11
Software		Atlas.ti (Scientific Software Development GmbH, Berlin, Version 9)	11
Participant checking		Participants did not provide feedback on the findings. Participants will receive the study manuscript after publication.	10
Reporting			
Quotations presented		Quotations are presented in the result section to illustrate the findings and are identified by participant number.	Results section
Data and findings consistent		There is consistency between the data presented and the findings.	Results section
Clarity of major themes		Major themes are clarified in the results section.	Results section
Clarity of minor themes		Minor themes are described in the results section and discussed in the discussion section.	Results and discussion section
